# Response of miR156-*SPL* Module during the Red Peel Coloration of Bagging-Treated Chinese Sand Pear (*Pyrus pyrifolia* Nakai)

**DOI:** 10.3389/fphys.2017.00550

**Published:** 2017-08-07

**Authors:** Minjie Qian, Junbei Ni, Qingfeng Niu, Songling Bai, Lu Bao, Jianzhao Li, Yongwang Sun, Dong Zhang, Yuanwen Teng

**Affiliations:** ^1^Department of Horticulture, Zhejiang University Hangzhou, China; ^2^The Key Laboratory of Horticultural Plant Growth, Development and Quality Improvement, Ministry of Agriculture of China Hangzhou, China; ^3^Zhejiang Provincial Key Laboratory of Integrative Biology of Horticultural Plants Hangzhou, China; ^4^College of Horticulture, Northwest A&F University Yangling, China

**Keywords:** pear, anthocyanin, red color pigmentation, miR156, *SQUAMOSA PROMOTER BINDING PROTEIN-LIKE* (SPL) genes

## Abstract

MicroRNA156 is an evolutionarily highly conserved plant micro-RNA (miRNA) that controls an age-dependent flowering pathway. miR156 and its target *SQUAMOSA PROMOTER BINDING PROTEIN-LIKE* (*SPL*) genes regulate anthocyanin accumulation in plants, but it is unknown whether this process is affected by light. Red Chinese sand pear (*Pyrus pyrifolia*) fruits exhibit a unique coloration pattern in response to bagging treatments, which makes them appropriate for studying the molecular mechanism underlying light-induced anthocyanin accumulation in fruit. Based on high-throughput miRNA and degradome sequencing data, we determined that miR156 was expressed in pear fruit peels, and targeted four *SPL* genes. Light-responsive elements were detected in the promoter regions of the miR156a and miR156ba precursors. We identified 19 *SPL* genes using the “Suli” pear (*Pyrus pyrifolia* Chinese White Pear Group) genome database, of which seven members were putative miR156 targets. The upregulated expression of anthocyanin biosynthetic and regulatory genes and downregulated expression of *PpSPL2, PpSPL5, PpSPL7, PpSPL9, PpSPL10, PpSPL13, PpSPL16, PpSPL17*, and *PpSPL18* were observed in pear fruits after bags were removed from plants during the anthocyanin accumulation period. Additionally, miR156a/ba/g/s/sa abundance increased after bags were removed. Yeast two-hybrid results suggested that PpMYB10, PpbHLH, and PpWD40 could form a protein complex, probably involved in anthocyanin biosynthesis. Additionally, PpSPL10 and PpSPL13 interacted with PpMYB10. The results obtained in this study are helpful in understanding the possible role of miR156 and its target *PpSPL* genes in regulating light-induced red peel coloration and anthocyanin accumulation in pear.

## Introduction

miR156 is one of the most abundant and evolutionarily conserved micro-RNAs (miRNAs) in plants, and affects diverse aspects of plant growth and development by regulating *SQUAMOSA PROMOTER BINDING PROTEIN-LIKE* (*SPL*) genes (Poethig, 2010; Huijser and Schmid, 2011). miR156 and the *SPL* genes are down- and up-regulated as plants develop, respectively (Wu and Poethig, 2006). In *Arabidopsis thaliana*, 11 out of the 17 *SPL* genes are targeted by miR156, including *SPL3, SPL4*, and *SPL5*, which form one group. Constitutive overexpression of miR156-resistant *SPL3* (*rSPL3*), *rSPL4*, or *rSPL5* results in early flowering (Cardon et al., 1997; Wu and Poethig, 2006). Additionally, SPL3 is a direct upstream activator of three transcription factors (i.e., LFY, FUL, and AP1) that are floral meristem identity genes (Yamaguchi et al., 2009). A second group includes *SPL9* and *SPL15*, which promote the juvenile-to-adult phase transition (Schwarz et al., 2008; Wang et al., 2008). The expression of a miR156-resistant *SPL9* (*rSPL9*) causes the extremely early flowering in plants (Wang et al., 2009; Wu et al., 2009). *SPL9* and *SPL15* also influence the characteristic appearance of abaxial trichomes on adult leaves (Wu et al., 2009), and ensure plants are capable of flowering in response to an appropriate photoperiod (Schwarz et al., 2008). A third group of *SPL* genes includes *SPL2, SPL10*, and *SPL11*, which have a minor role in vegetative phase changes in addition to their major role in early embryonic patterning *via* a redundant regulation of the embryonic morphogenesis-to-maturation phase transition (Nodine and Bartel, 2010).

Besides endogenous developmental cues, miR156-SPL module could also perceive various external stimuli as input signals for developmental and morphological adaptive changes (Wang and Wang, 2015). In Arabidopsis, high level of CO2 accelerates flowering probably by decreasing and increasing the level of miR156 and *SPLs*, respectively (May et al., 2013). Ambient temperature is also an input signal for miR156-SPL model as overexpression of miR156 causes delayed flowering at lower ambient temperature (16°C), associated with the down-regulation of *SPL3, FT*, and *FRUITFULL* expression (Kim et al., 2012). Additionally, miR156-SPL module is regulated by abiotic stresses, including Phosphate Deficiency (Hsieh et al., 2009), salt stress (Ding et al., 2009), and drought stress (Sun et al., 2012).

The miR156-SPL module was recently reported to be involved in regulating anthocyanin biosynthesis in plants (Gou et al., 2011; Cui et al., 2014). Anthocyanin is the main pigment in flowers and fruits, and is responsible for characteristic reddish, bluish, and purple hues. Anthocyanin biosynthesis involves several structural genes, including *PAL* (phenylalanine ammonia lyase), *CHS* (chalcone synthase), *CHI* (chalcone isomerase), *F3H* (flavanone 3-hydroxylase), *DFR* (dihydroflavonol 4-reductase), *ANS* (anthocyanidin synthase), and *UFGT* (UDP-glucose:flavonoid 3-O-glucosyltransferase) (Winkel-Shirley, 2001). Anthocyanin biosynthesis is regulated by numerous transcription factors, including MYB, bHLH, and WD40, which form a MYB/bHLH/WD40 complex that mediates the expression of anthocyanin biosynthetic genes in diverse species including Arabidopsis (Broun, 2005), Chinese bayberry (Liu et al., 2013), and strawberry (Schaart et al., 2013). Regulation of anthoycanin accumulation has been well studied in fruit crops. In litchi, *UFGT* and *LcMYB1* are the two key genes regulating anthocyanin biosynthesis and finally determining the pericarp color (Zhao et al., 2012; Lai et al., 2014). Delphinidin-derived anthocyanin is accumulated in the fruits of Lycium ruthenicum but not in the fruits of Lycium barbarum, which might be controlled by the expression patterns of both regulatory and structural genes and the transcriptional ratio of branch-node structural genes *F3*′*5*′*H*/*F3*′*H* (Zeng et al., 2014). In apple, a rearrangement in the upstream regulatory region of the *MYB10* is responsible for increasing the level of anthocyanin throughout the plant to produce a phenotype with red foliage and red fruit flesh (Espley et al., 2009). Anthocyanin biosynthesis is also regulated by other factors, such as sugar. In Arabidopsis, sucrose treatment could strongly up-regulated the flavonoid and anthocyanin biosynthetic pathways, thus affecting flavonoid and anthocyanin content (Solfanelli et al., 2006). In radish, exogenous glucose, fructose, or sucrose promotes anthocyanin accumulation in hypocotyl by inducing expression of the biosynthetic genes including *CHS* and *ANS* (Hara et al., 2003). Analyses of wildtype, miR156-upregulated (*Pro35S:MIR156*), and miR156-downregulated (*Pro35S:MIM156*) *A. thaliana* lines revealed that increased miR156 abundance promotes anthocyanin accumulation. Additionally, at least one of the miR156 target genes (i.e., *SPL9*) encodes a protein which can negatively regulate anthocyanin biosynthesis through disrupting the MYB/bHLH/WD40 complex by competing with bHLH for the binding of MYB (Gou et al., 2011). Under salt and mannitol stress conditions, SPL9 decreases anthocyanin accumulation by repressing the expression of *DFR* (Cui et al., 2014). In the blood-fleshed peach cultivar “Dahongpao” and the white-fleshed cultivar “Baifeng”, SPL1 represses the transactivation activity of the BL–PpNAC1 heterodimer, which subsequently inhibits *PpMYB10* expression, leading to limited accumulation of anthocyanins (Zhou et al., 2015). Additionally, miR172 abundance is regulated by miR156 and its targets *SPL* genes (Wu et al., 2009; Jung et al., 2011), and miR172 can be directly activated by *SPL9* and *SPL10* (Wu et al., 2009), indicating the possibility of miR172 participating in regulating anthocyanin biosynthesis in plants. These results reveal that the regulation of miR156/SPL modules during anthocyanin biosynthesis is affected by genotype and abiotic stress. There is currently little information regarding whether this regulatory pathway is influenced by light, which is one of the most important environmental factors required for anthocyanin accumulation in various plant species. COP1 and HY5 are the two key proteins involved in light-induced anthocyanin accumulation in fruit. COP1 is an ubiquitin E3 ligases, which is necessary for the ubiquitination and degradation of MdMYB1 protein in the dark and therefore negatively regulates light-induced anthocyanin biosynthesis in apple (Li et al., 2012). However, under UV-B irradiation, the expression of *MdCOP1* is induced, which activates MdHY5 signaling by binding to the promoter regions of *MdMYBs*, and finally leads to the red coloration of apple peels (Peng et al., 2012). However, besides light signal transduction pathway, more other pathways which regulate light-induced anthocyanin biosynthesis in fruit need to be defined.

Pear (*Pyrus* spp.) is one of the most dominant temperate fruit crops worldwide. Anthocyanin accumulation in pear is considerably different than that of other important fruit species, including grape and apple. Grape rapidly softens at the beginning of the second growth cycle (the growth curve of grape is double-sigmoidal pattern), many weeks before ripeness, and the onset of rapid softening is called “veraison” by viticulturists. After varaison, red colored grape cultivars start accumulating anthocyanin, and the skin color is changed from green to red (Coombe, 1973). Apple shows two peaks of anthocyanin concentration during development: the fruitlet of apple is red, then the red color disappears, and anthocyanin is accumulated again and peaked toward maturity (Steyn et al., 2005). For pear, the fruit pigmentation patterns differ between the two main commercial pear species, Chinese sand pears (*Pyrus pyrifolia* Nakai) and European pears (*Pyrus communis* L.). Unlike red Chinese sand pears, in which anthocyanin accumulation peaks in mature fruit (Huang et al., 2009), European pears exhibit a decreased red coloration as fruits mature. The highest anthocyanin levels in European pears occur about midway between anthesis and harvest (Steyn et al., 2005). Chinese sand pears and European pears also respond differently to bagging treatments and postharvest UV-B/visible light irradiation. Fruit bagging and postharvest UV-B/visible light irradiation are considered attached and detached treatments to induce anthocyanin biosynthesis and improve fruit color. Fruit bagging has been used in apples (Arakawa, 1988; Bai et al., 2016) and Asian pears (Huang et al., 2009; Qian et al., 2013). Postharvest irradiation has been widely applied to apples (Ubi et al., 2006), Asian pears (Qian et al., 2013; Sun et al., 2014), grapes (Li et al., 2009), and cherries (Kataoka et al., 2005). Ten days after bags are removed from plants or 10 days after the start of postharvest irradiation, Chinese sand pears appear red (i.e., accumulated anthocyanin), while anthocyanin is almost undetectable in European pears (Qian et al., 2013). The distinct pigmentation pattern in response to bagging treatments makes Chinese sand pear appropriate for studying the molecular mechanism regulating light-induced anthocyanin accumulation in fruits. However, it is currently unknown whether miR156/SPL modules affect this process in pear plants.

Different family members of miR156 and *SPL* genes have been identified (Cardon et al., 1999; Yu et al., 2015), and the abundance of miR156 exhibits tissue specificity (Xia et al., 2012; Yu et al., 2015). Pear miRNAs have been identified in fruit flesh and buds by high-throughput sequencing (Wu et al., 2014; Niu et al., 2016). In this study, we used miRNA sequencing, degradome analysis, and a genome-wide identification of *SPL* gene family members, to select miR156 and *SPL* members that might participate in the light-induced anthocyanin biosynthesis in pear fruit peel. Furthermore, we searched for light-responsive elements in the promoter region of MIR156 genes (i.e., pre-miR156). We also examined the expression patterns of these potential miR156 and *SPL* genes after bagging treatments, and investigated the interaction between SPLs and the MYB/bHLH/WD40 transcription factor complex.

## Materials and methods

### Plant materials, treatments, and total RNA extraction

Red Chinese sand pear (cultivar “Meirensu”) fruits were obtained from a commercial orchard in Kunming, Yunnan, China. Ten mature trees that were similar in size and uniformly exposed to sunlight were selected. We bagged 80 fruitlets (the vertical diameter of fruit was around 2 cm) per tree 40 days after full bloom with double-layered yellow–black paper bags [Kobayashi (Qingdao) Co., Ltd., China]. Half of the fruits were re-exposed to sunlight after the bags were removed 10 days before harvest (the total bagging period lasted for about 3 months). The fruits that remained bagged served as control samples. Fruits were harvested 0, 1, 3, and 6 days after bag removal (DABR), stored on dry ice, and transported to the lab as quickly as possible. The skin from 10 individual fruits were pooled, instantly frozen in liquid nitrogen, and stored at −80°C for assays.

### Small-RNA library construction, sequencing, and data analysis

Total RNA from re-exposed and control fruit peels collected at 0, 1, 3, and 6 DABR was isolated using a CTAB method (Qian et al., 2014a). After ligation of 3′- and 5′- adapters, and RT-PCR amplification, small RNA (sRNA) library was size fractioned by PAGE gel. A mixed sRNA library was constructed and sequenced using the Illumina Genome Analyzer IIx (Illumina, Inc., Santa Clara, CA, USA). After filtering and adapter cutting, the clean tags were used by BLASTall to search the GenBank and Rfam 10.0 databases (Kozomara and Griffiths-Jones, 2014) to detect rRNAs, scRNAs, snoRNAs, snRNAs, and tRNAs, which were subsequently removed. The remaining sRNA tags were aligned with mRNA sequences to identify any degraded mRNA fragments, which were eliminated (http://peargenome.njau.edu.cn/default.asp?d=1&m=1) (Wu et al., 2013). The potential miRNAs were then mapped to the pear genome sequence (http://peargenome.njau.edu.cn:8004/default.asp?d=4&m=2) (Wu et al., 2013) using SOAP 2.20 (http://soap.genomics.org.cn/soapsplice.html), and their distribution on the genome was analyzed. Only sRNA tags that formed good stem-loop structures and had a miRNA/miRNA^*^ pair were considered as potential miRNAs. The secondary structure was predicted by RNAfold, and novel miRNAs were identified by MIREAP. The criteria used to identify candidate miRNAs were as previously described (Niu et al., 2013). Analyses of the high-throughput sequencing profiles were based on the number of reads. The miRNA expression levels were converted to transcripts per million normalized values as follows: normalized expression = actual miRNA count/total number of clean reads × 1,000,000. Raw data from sRNA sequencing was submitted to Sequence Read Archive (SRA ID: SRR5074534).

### Degradome library construction, data analysis, and target identification

Equal amounts of RNA from seven independent pear fruit peel libraries (re-exposed and control fruit peels collected at 0, 1, 3, and 6 DABR) were pooled to construct a degradome library. After 5′ adapter contaminants were eliminated and genome mapping was completed as described for the sRNA data, the degradome sequencing data were analyzed using the CleaveLand (version 3.0) (Addo-Quaye et al., 2009) program. The alignment score threshold was set to 4.5 for conserved and moderately conserved miRNAs and to 5 for novel and candidate miRNAs (Xia et al., 2012). The apple consensus gene set from AppleGFDB and the annotation information for miRNA target genes were retrieved from the Genome Database for Rosaceae (Zhang et al., 2013). Degradome data were normalized to transcripts per million values. Based on the number of degradome sequences and their abundance values, the cleavage sites of miRNA targets were classified into 5 categories as previous study (Xia et al., 2012): Category 0: > 1 raw read at the position, read abundance at position is equal to the maximum read abundance on the transcript, and there is only one maximum on the transcript. Category 1: > 1 raw read at the position, read abundance at position is equal to the maximum read abundance on the transcript, and there is more than one maximum position on the transcript. Category 2: > 1 raw read at the position, read abundance at position is less than the maximum but higher than the median read abundance for the transcript. Category 3: > 1 raw read at the position, read abundance at position is equal to or less than the median read abundance for the transcript. Category 4: only 1 raw read at the position. Raw data from degradome sequencing was submitted to Sequence Read Archive (SRA ID: SRR5074535).

### 5′-rapid amplification of cDNA ends

We completed 5′-rapid amplification of cDNA ends (RACE) using the SMARTer RACE cDNA Amplification Kit (Clontech, Palo Alto, CA, USA) to verify the miR156-mediated cleavage events identified during degradome sequencing. A 2-μg sample of mixed total RNA isolated from seven independent pear fruit peels (re-exposed and control fruit peels collected at 0, 1, 3, and 6 DABR) was ligated with 5′ RNA adapters at room temperature. All polymerase chain reaction (PCR) products were inserted into the pMD18-T vector (Takara, Dalian, China) and sequenced. Specific primers were designed for nested PCR (Supplementary Table S1 in Supplementary File 1).

### Gene ontology analysis

An additional analysis involving the Gene Ontology (GO) databases was completed to more thoroughly characterize the functions of miRNA targets. Blast2GO was used to store and manage information from the GO databases (https://www.blast2go.com). All targets were identified using BLASTx searches against the GO protein database. The GO analyses were completed using a query search of the GO databases.

### Identification and annotation of pear *SQUAMOSA promoter binding protein (SBP)-box* genes

The hidden Markov model (HMM) profile of the SBP domain (Accession no. PF03110) was downloaded from the Pfam database (http://www.sanger.ac.uk). This domain was then used as a query for BLASTP searches of the GenBank non-redundant protein database and the Pear Genome Database (http://peargenome.njau.edu.cn:8004/default.asp?d=4&m=2) (Wu et al., 2013). All hits with an *E* < 0.01 were collected. The non-redundant protein sequences encoded by putative *PpSPL* genes were manually checked for the presence of the SBP domain, and genes without a complete SBP domain were eliminated. All putative *PpSPL* genes were confirmed again by cloning and sequencing, using a mixed cDNA template obtained from different tissues of “Meirensu” plants, including young fruits, young leaves, petals, young stems, and the skin of mature fruit. Primer details are provided in Supplementary Table S1 in Supplementary File 1. The *PpSPL* genes were named according to their gene ID order in the pear genome.

### Sequence alignments and phylogenetic analyses

Multiple sequences were aligned using DNAMAN software (version 5.2). The sequence logo was obtained using the online WebLogo platform (http://weblogo.berkeley.edu). Phylogenetic trees were constructed using the neighbor-joining method of the MEGA 4.0 program (Tamura et al., 2007), and the bootstrap test was replicated 1,000 times. The miR156 target sites were identified using sequence alignments and manual analyses.

### Extraction and measurement of total anthocyanin

Total anthocyanin content was measured using a pH differential method and was calculated as mg cyanidin-3-galactoside per 100 g fresh tissue (mg Cy-3-gal/100 g FW) (Dussi et al., 1995). Fruit tissue (1 g) was mixed with methanol and 0.01% HCl, and then centrifuged at 18,514 g for 20 min at 4°C. The absorbance (at 510 nm and 700 nm) of a 100-μL extract in buffers (pH 1.0 and 4.5) was measured using a DU800 spectrophotometer (Beckman Coulter, Fullerton, CA, USA). Total anthocyanin content was calculated as follows: A = [(A_510_− A_700_) pH_1.0_− (A_510_− A_700_) pH_4.5_], with a cyanidin-3-galactoside molar extinction coefficient of 3.02 × 10^4^.

### Quantitative real-time polymerase chain reaction analysis

Total RNA was extracted using a modified CTAB method (Qian et al., 2014a). The total RNA sample was treated with DNase I to remove any contaminating genomic DNA, and then quantified. First-strand cDNA was synthesized from 4 μg DNA-free RNA using a Revert Aid™ First-Strand cDNA Synthesis Kit (Fermentas, Glen Burnie, MD, USA). A 2-μL sample of 10-fold diluted cDNA was used as the template for gene cloning and quantitative real-time polymerase chain reaction (qPCR) analyses.

The qPCR mixture contained 10.0 μL SYBR Premix Ex Taq™ (Takara, Ohtsu, Japan), 0.4 μL each primer (10 μM), 2 μL cDNA, and 7.2 μL RNase-free water in a total volume of 20 μL. The PCR was completed using a LightCycler 1.5 instrument (Roche, Germany) with the following program: 95°C for 30 s; 40 cycles of 95°C for 5 s and 60°C for 20 s. A template-free control was included for each primer pair. The qPCR primers (Supplementary Tables S2, S3 in Supplementary File 1) were designed using Primer 3 software (http://frodo.wi.mit.edu/cgi-bin/primer3/primer3_www.cgi). All qPCR data were normalized using the threshold cycle value for the *Pyrus* species actin gene (*PyActin*, JN684184). Each sample was analyzed three times.

### Quantitative real-time polymerase chain reaction analysis of miR156 in pear

Total RNA was extracted using the pBiozol Total RNA Extraction Reagent (BioFlux, China). According to the instructions provided for the miRNA cDNA Synthesis Kit (Takara), 1 μg total RNA was polyadenylated by poly(A) polymerase. The poly(A)-tailed total RNA was reverse-transcribed by PrimeScript® RTase with a universal adapter primer (containing oligo-dT). The qPCR analysis was completed using SYBR® Premix Ex Taq II (Perfect Real Time) (Takara) and a LightCycler 1.5 instrument. Each sample was analyzed three times, and 5S rRNA was used as the internal control gene (Wu et al., 2014; Niu et al., 2016). The qPCR primers are listed in Supplementary Table S4 in Supplementary File 1.

### Yeast two-hybrid assay

A yeast two-hybrid assay was conducted using the Matchmaker™ Gold Yeast Two-Hybrid System (Clontech). We used the pGADT7 vector, which contains the GAL4 activation domain, and the pGBKT7 vector, which carries the GAL4 DNA binding domain. The gene open reading frames were inserted into the multiple cloning sites of pGBKT7 and pGADT7 using the In-Fusion® HD Cloning Kit (Clontech) to obtain the gene-BD and gene-AD plasmids, respectively. Details of the primers used during the yeast two-hybrid assay to obtain the open reading frames are listed in Supplementary Table S5 in Supplementary File 1. The gene-BD plasmid was inserted alone or together with the gene-AD plasmid into the Y2HGold yeast strain according to a polyethylene glycol/lithium acetate method. The autoactivation of the colonies transformed with the gene-BD plasmid was tested on synthetically defined (SD) medium lacking tryptophan, but supplemented with 5-bromo-4-chloro-3-indolyl α-D-galactopyranoside (X-α-Gal) and aureobasidin A (AbA). The co-transformed colonies were selected on SD medium lacking leucine and tryptophan (i.e., double dropout medium), and screened for growth on quadruple dropout SD medium (i.e., lacking adenine, histidine, leucine, and tryptophan) supplemented with X-α-Gal and AbA to verify positive interactions.

### Statistical analysis

After running ANOVA, LSDs (α = 0.05) were calculated for mean separations of anthocyanin concentration using the Data Processing System (DPS, version 3.01, Zhejiang University, Hangzhou, China). Differences of gene expression were statistically evaluated by the analysis of variance and Tukey's test using SPSS 19.0 (SPSS, Chicago, IL, USA). Probability values of <0.05 were considered statistically significant.

## Results

### Genome-wide identification of pear miRNAs and their targets, and analysis of promoter elements of miR156 precursors

A total of 13,954,538 raw reads were acquired through high-throughput sequencing of sRNA from pear fruit peels of bagged plants. Corrupted adapter sequences (e.g., missing 3′ adapter), sequences shorter than 17 bases or 25 bases after removing the 3′ adapter, and junk reads were eliminated. The remaining 9,915,560 clean reads were used for predicting miRNAs (Supplementary Table S6 in Supplementary File 1). A total of 167 conserved, 34 moderately conserved, and 21 novel miRNAs were identified in the sRNA library (Supplementary Tables S7, S8 in Supplementary File 1). Among the detected miRNAs, 26 miR156 members were identified, including some that were highly expressed in fruit peels such as miR156c (424 reads). Some members were detected at very low levels, including miR156sd (1 read) and miR156bd-3p (1 read). In addition, all the novel miRNAs were highly expressed in fruit peel, with the raw reads from 125 to 1,866 (Supplementary Tables S8 in Supplementary File 1).

For the degradome sequence analysis, ~17.71 million raw reads were obtained, and 9,052,407 unique reads were ultimately generated. These reads were subsequently screened and analyzed with CleaveLand 2.0 (Addo-Quaye et al., 2009). A total of 130 targets with cleavage sites grouped into five categories (i.e., 0–4) were identified in fruit peels (Supplementary Table S9 in Supplementary File 1), with 86 targets having GO numbers (Supplementary Table S10 in Supplementary File 1). Many of the identified targets for the conserved pear miRNAs were transcription factors, such as the auxin response factor targeted by miR160, ethylene-responsive transcription factor targeted by miR172, NAC domain protein targeted by miR164, and a GRAS family transcription factor targeted by miR171 (Supplementary Table S10 in Supplementary File 1). Among 21 novel miRNAs, miRC9 and miRC10 targeted 4 (Pbr008410.2, Pbr008785.1, Pbr037485.1, and Pbr019133.1) and 2 (Pbr001375.1 and Pbr002864.1) genes, respectively, and two of these target genes had GO numbers, with Pbr008410.2 and Pbr001375.1 encoding a homeobox-leucine zipper protein and glutamate dehydrogenase, respectively (Supplementary Tables S9, S10 in Supplementary File 1). Notably, four pear *SPL* genes were targeted by miR156. Pbr019232.1, which is homologous to *A. thaliana SPL9*, was targeted by miR156ba. Pbr020183.1 (*SPL6* homolog), Pbr037448.1, and Pbr038099.1 (*SPL13* homolog) were targeted by miR156a/g/s/sa (Supplementary Table S10 in Supplementary File 1).

Due to the results from sRNA and degradome sequencing, miR156a and miR156ba were chosen as two examples of miR156s, and the genomic region 1,500 bp upstream of pre-miR156a and pre-miR156ba was considered the promoter region and was analyzed using the PlantCARE online program (http://bioinformatics.psb.ugent.be/webtools/plantcare/html/). We identified 16 light-responsive elements in the pre-miR156a promoter (i.e., two Box 4 elements, six G-boxes, one GA-motif, one GAG-motif, two GT1-motifs, one I-box, one MRE, and two sp1 elements) (Table 1). The pre-miR156ba promoter contained one AE-box, two Box 4 elements, seven G-boxes, one GAG-motif, three GT1-motifs, one TCCC, and two sp1 elements (Table 1).

**Table 1:** *Cis*-acting elements potentially associated with promoter region of pre-miR156.

**Motif**	**Strand**	**Distance from precursor**	**Sequence**	**Function**
**miR156a**				
AE-box	−	1,300	AGAAACAA	part of light-responsive module
Box 4	+	390	ATTAAT	part of a conserved DNA module involved in light responsiveness
	−	1,485	ATTAAT	
G-Box	+	647	CACGTA	cis-acting regulatory element involved in light responsiveness
	−	968	CACGTA	
	+	658	CACGTA	
G-Box	−	647	TACGTG	cis-acting regulatory element involved in light responsiveness
	−	967	TAACACGTAG	
	−	658	TACGTG	
	+	968	TACGTG	
GAG-motif	−	725	AGAGATG	part of light-responsive element
GT1-motif	−	43	GGTTAA	light-responsive element
	−	939	AATCCACA	
	+	739	GGTTAAT	
Sp1	+	858	CC(G/A)CCC	light-responsive element
	−	1,030	CC(G/A)CCC	
**miR156ba**				
Box 4	+	362	ATTAAT	part of a conserved DNA module involved in light responsiveness
	−	1,449	ATTAAT	
G-Box	+	678	CACGTT	cis-acting regulatory element involved in light responsiveness
	−	1,010	CACGTA	
G-Box	−	649	CACATGG	cis-acting regulatory element involved in light responsiveness
	−	1,009	TAACACGTAG	
	+	678	CACGTT	
	+	1,010	TACGTG	
GA-motif	−	235	AAGGAAGA	part of light-responsive element
GAG-motif	−	903	AGAGAGT	part of light-responsive element
GT1-motif	+	773	GGTTAAT	light-responsive element
	−	981	AATCCACA	
I-box	−	430	ATGATATGA	part of light-responsive element
MRE	−	780	AACCTAA	MYB binding site involved in light responsiveness
Sp1	+	876	CC(G/A)CCC	light-responsive element
	+	971	CC(G/A)CCC	
TCCC-motif	−	368	TCTCCCT	part of light-responsive element

### Genome-wide identification of *PpSPL* genes

**Figure 1:**
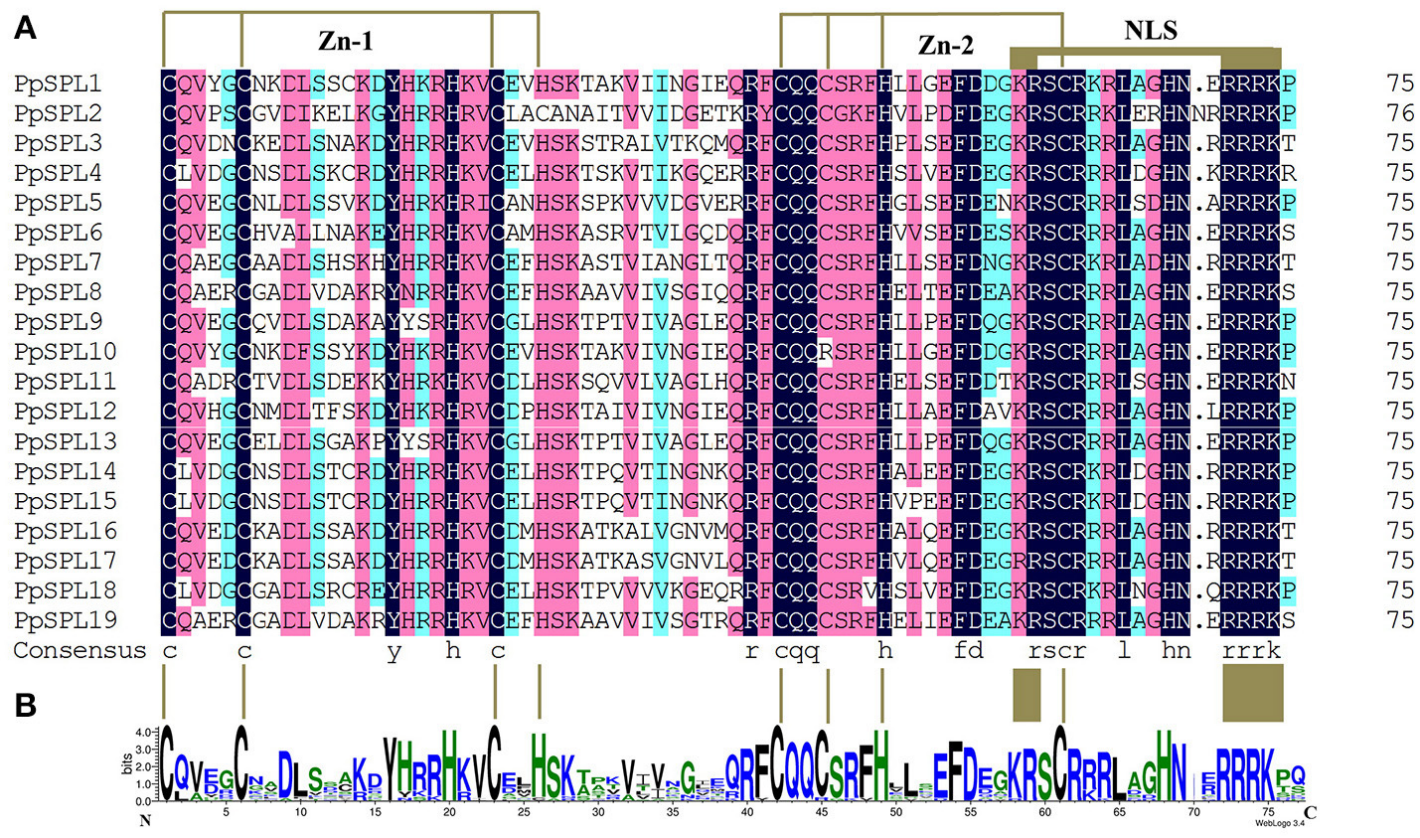
Alignment of the SBP domains of PpSPL proteins. **(A)** Multiple SBP domain sequences from pear PpSPL proteins were aligned using DNAMAN software. Two conserved zinc finger structures (i.e., Zn-1 and Zn-2) and the nuclear localization signal are indicated. **(B)** Sequence logo of the SBP domain of PpSPL proteins. The overall height of each stack represents the degree of conservation at this position, while the height of the letters within each stack indicates the relative frequency of the corresponding amino acids.

**Figure 2:**
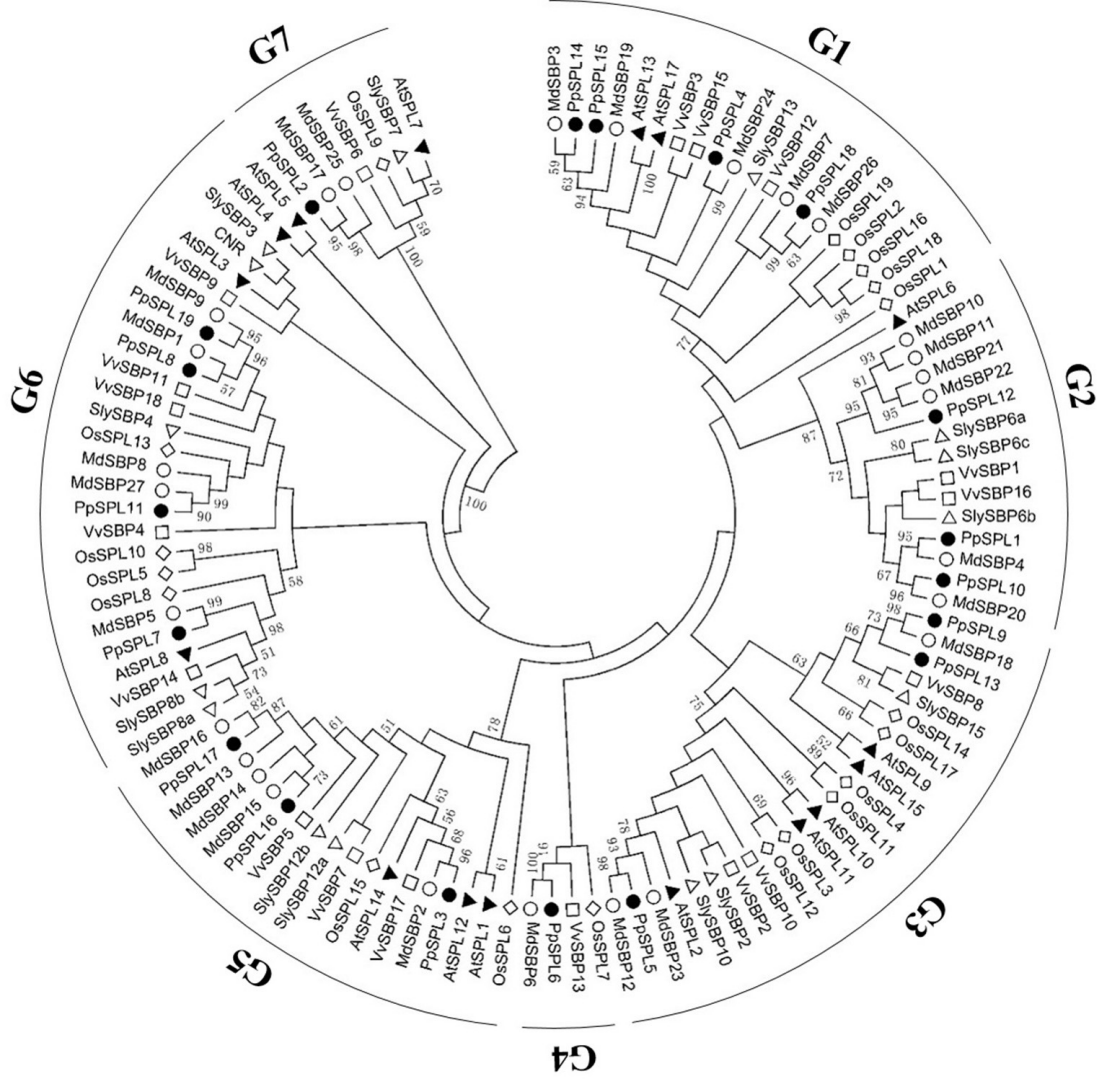
Phylogenetic analysis of SPLs from pear and other plant species. A phylogenetic tree was constructed using SBP domain protein sequences from pear (PpSPL, marked with black dots), apple (MdSBP, marked with white dots), grape (VvSBP, marked with white squares), rice (OsSPL, marked with white rhombuses), tomato (CNR or SlySBP, marked with white triangles), and *Arabidopsis thaliana* (AtSPL, marked with black triangles). The symbols for *Arabidopsis thaliana* and tomato genes are inverted in the right and left halves of the figure. *PpSPL1, PpSPL4, PpSPL5, PpSPL6, PpSPL9, PpSPL10, PpSPL12, PpSPL13, PpSPL14, PpSPL15*, and *PpSPL18* are putative miR156 targets. The SBP domain sequences used to construct the phylogenetic tree as well as the accession numbers or locus IDs and data sources of the SBP-box genes from different species are listed in Supplementary Table S12 in Supplementary File 1.

We isolated 19 pear *PpSPL* genes, which were named based on the gene ID order in the pear genome (i.e., *PpSPL1–19*) (Supplementary Table S11 in Supplementary File 1). All PpSPL family members shared a conserved SBP domain, with two zinc finger-like structures (i.e., Zn-1 and Zn-2) and a highly conserved bipartite nuclear localization signal (NLS; Figure 1). To further study the evolutionary relationships among *PpSPL* genes and *SPL* genes from other plant species, we prepared a dataset of 95 *SPL* sequences from apple, grape, rice, tomato, and *A. thaliana*. The amino acid sequences of the SBP domains (75 amino acids) encoded by the *SPL* genes were used to construct a phylogenetic tree with the neighbor-joining algorithm (Figure 2). The plant SBP domains were clustered into seven distinct groups (i.e., G1–G7). The *PpSPL* family members were distributed over all seven cluster groups, but were most closely associated with apple genes. Additionally, *PpSPL9* and *PpSPL13* were homologs of *A. thaliana AtSPL9*, which negatively regulates anthocyanin biosynthesis.

### Genome-wide analysis of pear miR156 targets

**Figure 3:**
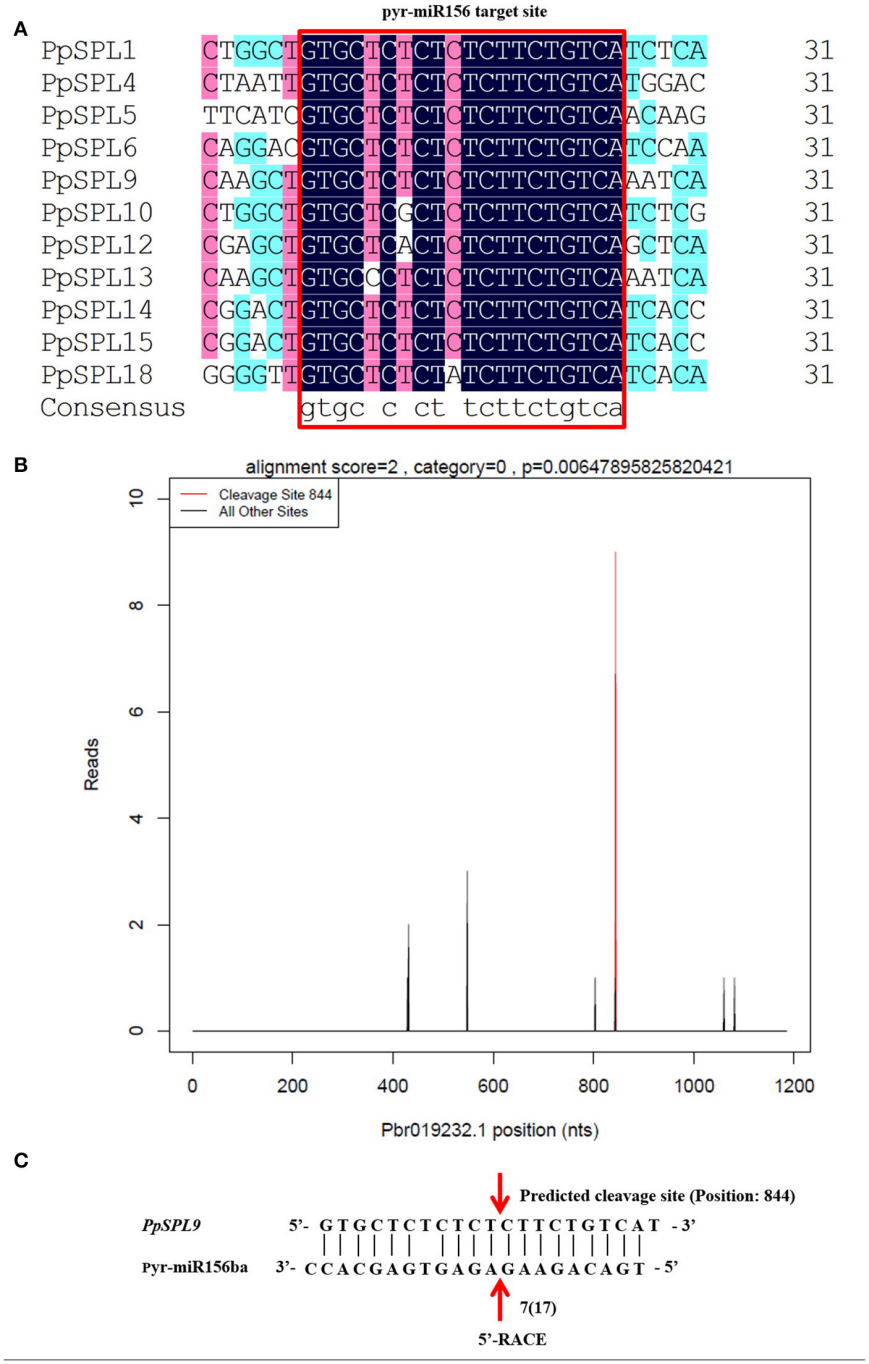
miR156 and its target *PpSPL* genes. **(A)** Alignment of multiple pyr-miR156 target site sequences from *PpSPL* genes using DNAMAN software. **(B)** T-plot indicating the cleavage events mediated by miR156ba in *PpSPL9* based on degradome sequencing results. **(C)** Cleavage site mediated by miR156ba in *PpSPL9*. The cleavage events identified during degradome sequencing were verified by 5′-rapid amplification of cDNA ends (5′-RACE). 17 colonies were sequenced, and 7 of them corresponded to the cleavage site of position 844.

Based on analyses of the degradome, *PpSPL9* (Pbr019232.1), *PpSPL10* (Pbr020183.1), *PpSPL15* (Pbr037448.1), and *PpSPL18* (Pbr038099.1) were targeted by miR156 in fruit peels (Figure 3A; Supplementary Table S10 in Supplementary File 1). Additionally, the cleavage site of the miR156ba-targeted *PpSPL9* gene was also confirmed by 5′-RACE nested PCR (Figure 3B). Some miR156-targeted *PpSPL* genes may have been missed during the analysis of the degradome because of inactive genes or low levels of target gene expression in mature pear fruit peels. To assess this possibility, we completed target prediction analyses for all identified *PpSPL* genes based on sequence complementarity with miR156. We determined that an additional seven *PpSPL* genes (i.e., *PpSPL1, PpSPL4, PpSPL5, PpSPL6, PpSPL12, PpSPL13*, and *PpSPL14*) were putative miR156 targets (Figure 3A). A total of 11 *PpSPL* genes were potentially regulated by miR156. Interestingly, all 11 miR156-targeted *PpSPL* genes were clustered into G1–G4 in the phylogenetic tree (Figure 2).

### Anthocyanin accumulation, and expression profiles of anthocyanin biosynthetic and regulatory genes, *PpSPL* genes, and miR156 genes after bags were removed from “Meirensu” pear plants

**Figure 4:**
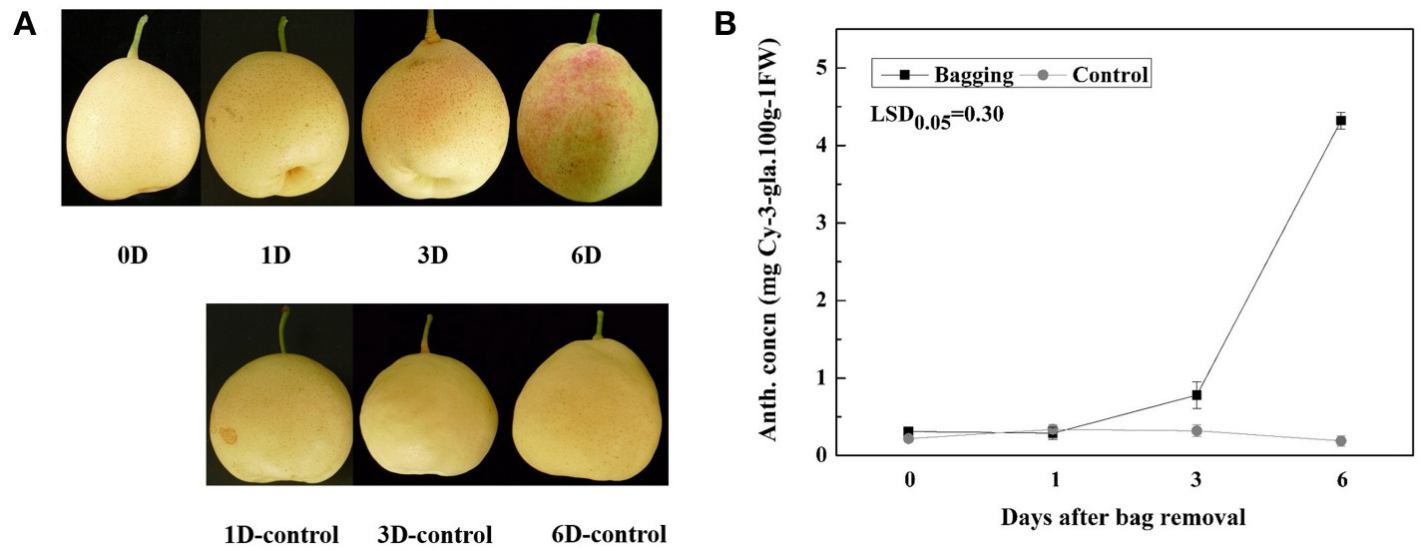
**(A)** Peel color of “Meirensu” at different stages after bags were removed from plants. **(B)** Anthocyanin concentration (anth. concn.) of “Meirensu” pear fruit skin at different stages after bags were removed from plants. Each value represents the mean ± standard error of three biological replicates.

**Figure 5:**
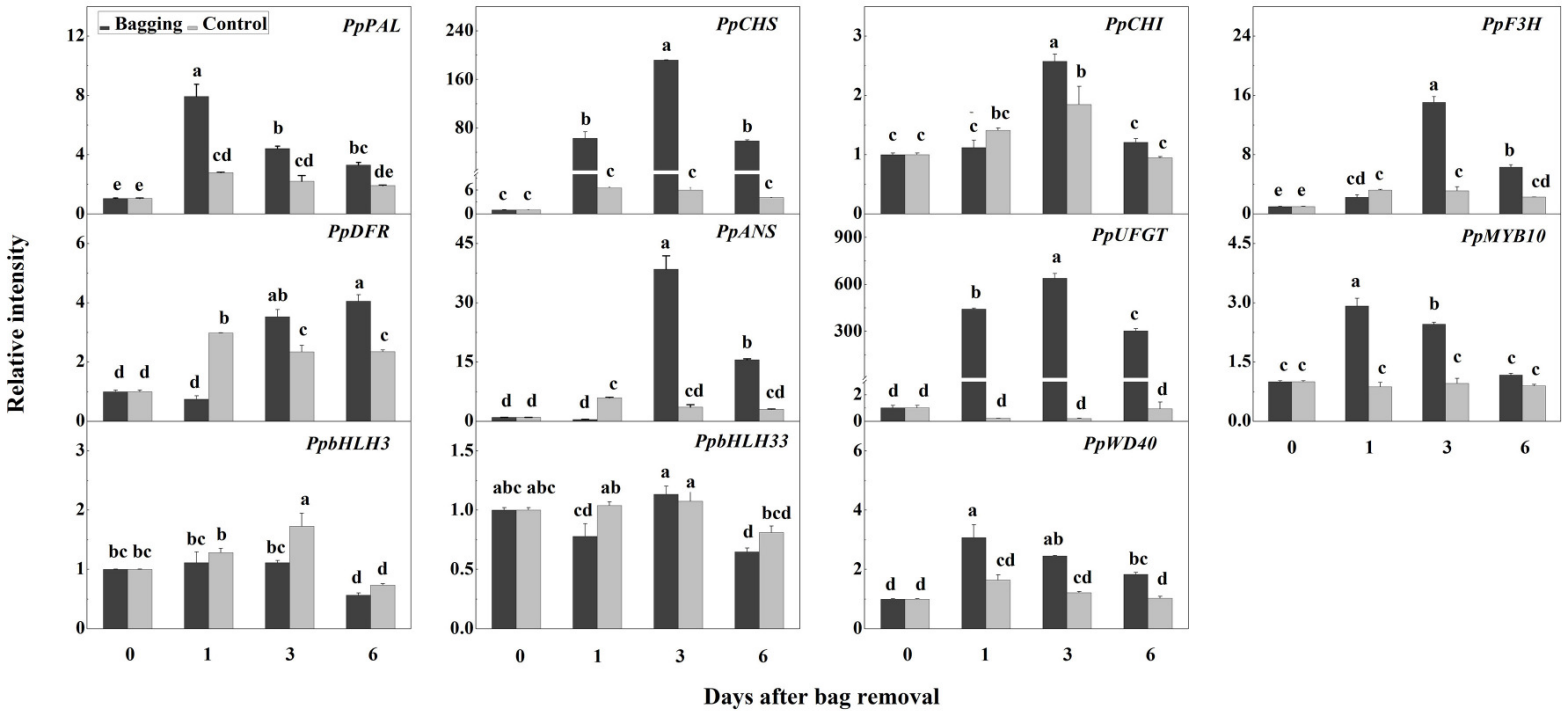
Expression of anthocyanin biosynthetic genes and regulatory genes at different stages after bags were removed from plants. Each value represents the mean ± standard error of three biological replicates.

Anthocyanin started to accumulate in pear fruit peels at 3 DABR, and reached about 4.5 mg Cy-3-gal/100 g FW at 6 DABR, while no anthocyanin was detected in control fruit peels (Figures 4A,B). Additionally, after bags were removed, the expression of most anthocyanin biosynthetic genes was upregulated, with peak levels at 1 DABR for *PpPAL*, 3 DABR for *PpCHS, PpCHI, PpF3H, PpANS*, and *PpUFGT*, and 6 DABR for *PpDFR* (Figure 5). Notably, the *PpCHS, PpANS*, and *PpUFGT* transcript levels at 3 DABR were about 200-, 40-, and 600-fold higher than the transcript levels at 0 DABR, respectively (Figure 5). Among the transcription factors, the expression levels of *PpMYB10* and *PpWD40* increased significantly, and peaked at 1 DABR (i.e., about a 3-fold increase; Figure 5).

**Figure 6:**
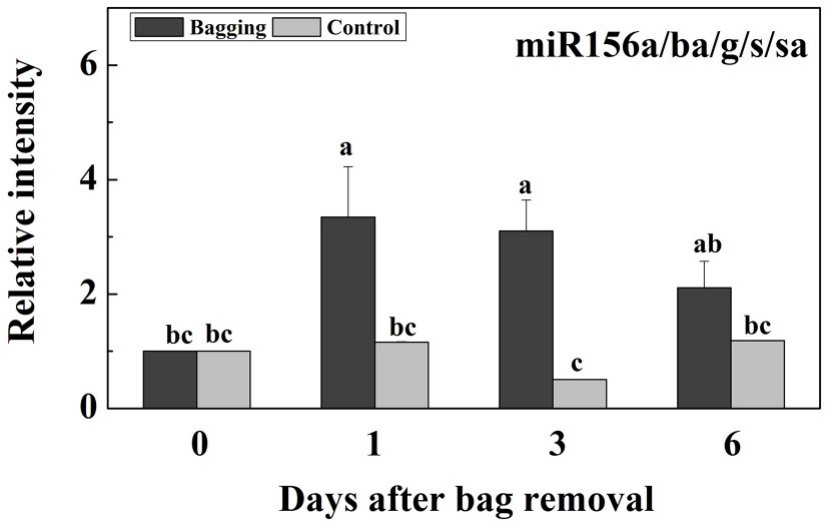
Expression of miR156a/ba/g/s/sa at different stages after bags were removed from plants. Each value represents the mean ± standard error of three biological replicates.

Of the miR156 members, miR156a/g/s/sa and miR156ba shared highly similar sequences. Therefore, a conserved primer was designed for analyzing the total expression of miR156a/g/s/sa and miR156ba. After bags were removed from plants, miR156a/ba/g/s/sa transcript abundance increased, peaking at 1–3 DABR (i.e., >3-fold increase) followed by a gradual decrease, but the expression level was still relatively high at 6 DABR (Figure 6). In contrast, miR156a/ba/g/s/sa transcript abundance in the control samples was low at all time points.

**Figure 7:**
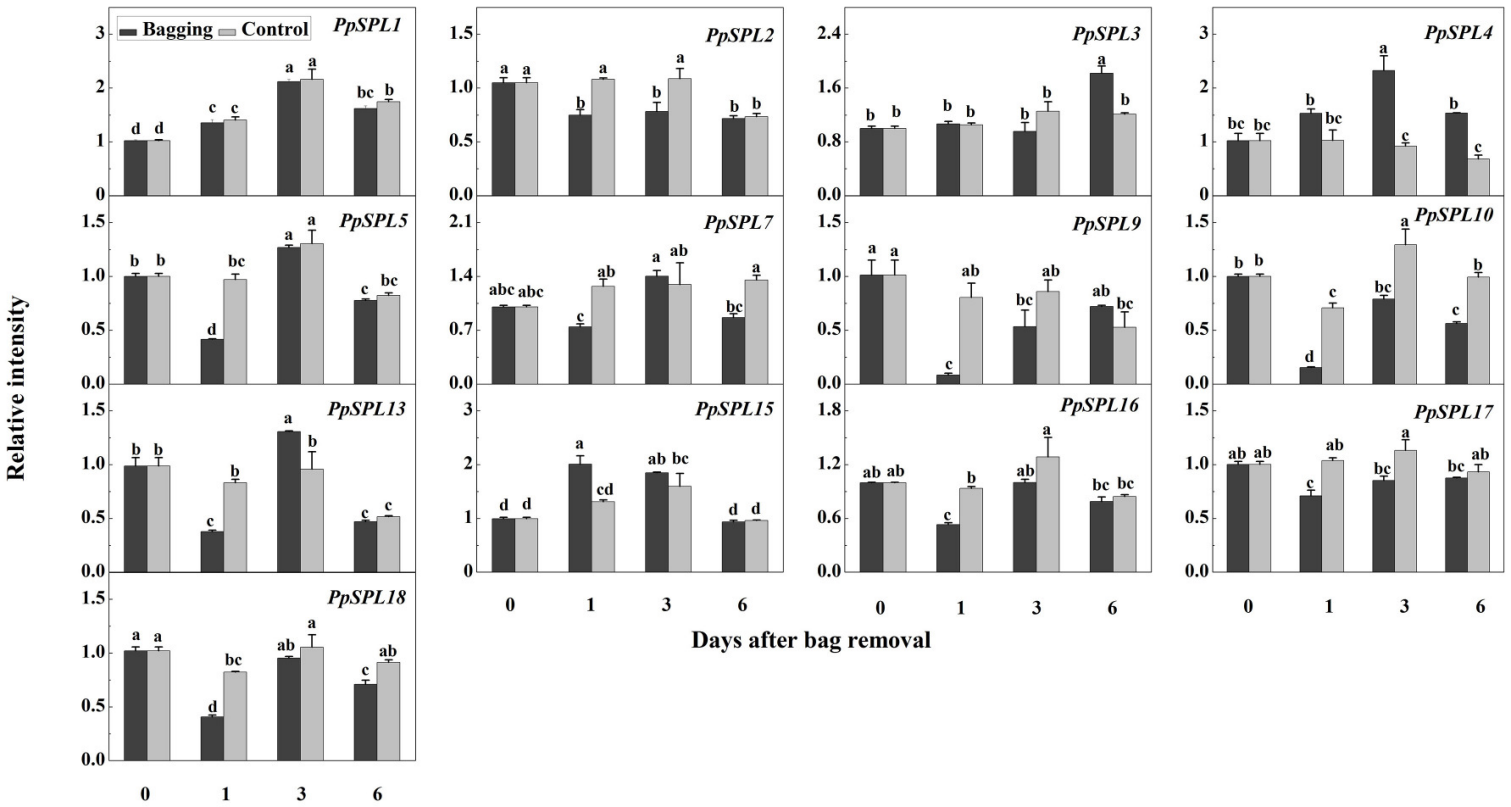
Expression of *PpSPL* genes at different stages after bags were removed from plants. Each value represents the mean ± standard error of three biological replicates.

We did not detect the expression of *PpSPL6, PpSPL8, PpSPL11, PpSPL12, PpSPL14*, and *PpSPL19* in mature “Meirensu” pear fruit peels. The expression of *PpSPL3, PpSPL4*, and *PpSPL15* was upregulated after bag removal, and peaked at 6, 3, and 1 DABR, respectively (Figure 7). The transcription of *PpSPL13* was decreased at 1DABR but upregulated at 3 DABR (Figure 7). The expression of *PpSPL2, PpSPL5, PpSPL7, PpSPL9, PpSPL10, PpSPL16, PpSPL17*, and *PpSPL18* was downregulated at 1 DABR, with the greatest decrease observed for *PpSPL9* and *PpSPL10* (Figure 7). Among these nine downregulated *SPL* genes, five were targeted by miR156, namely *PpSPL5, PpSPL9, PpSPL10, PpSPL13*, and *PpSPL18. PpSPL9* and *PpSPL13* were homologs of *A. thaliana SPL9*.

### Interactions among transcription factors related to anthocyanin biosynthesis

**Figure 8:**
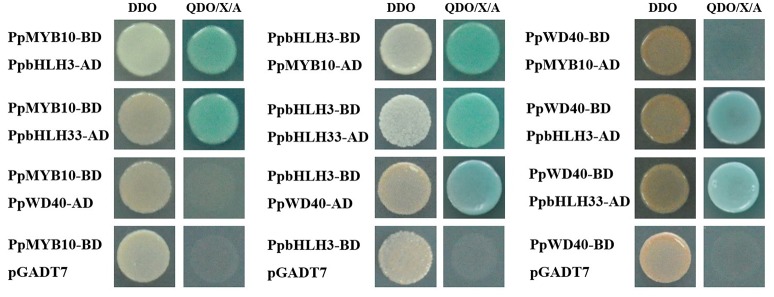
Yeast two-hybrid analysis of the physical interactions among transcription factors related to pear anthocyanin biosynthesis. DDO: double dropout synthetically defined (SD) medium (i.e., lacking leucine and tryptophan). QDO/X/A: quadruple dropout SD medium containing X-α-Gal and aureobasidin A (AbA) (i.e., SD medium lacking adenine, histidine, leucine, and tryptophan, but supplemented with X-α-Gal and AbA). The empty pGADT7 vector was used as a negative control.

Because anthocyanin biosynthesis involves three transcription factors (i.e., MYB, bHLH, and WD40), the interaction among these proteins was tested with a yeast two-hybrid assay. The *PpMYB10, PpbHLH3, PpbHLH33*, and *PpWD40* open reading frames were inserted into the pGBKT7 and pGADT7 vectors to obtain BD-bait and AD-prey protein complexes. We used SD medium lacking tryptophan, but supplemented with X-α-Gal and AbA, to verify the autoactivation of BD-bait protein complexes. PpMYB10-BD, PpbHLH3-BD, and PpWD40-BD were not autoactivated on medium containing 125 ng/ml AbA. However, PpbHLH33-BD was autoactivated on medium with 1,000 ng/ml AbA (data not shown), so it could not be analyzed by the yeast two-hybrid assay. Our results indicated that PpMYB10 and PpWD40 did not interact with each other, while both transcription factors interacted with PpbHLH3 and PpbHLH33 (Figure 8). These findings suggested that a MYB10/bHLH/WD40 protein complex exists in pear.

### Interactions among PpSPL proteins and transcription factors related to anthocyanin biosynthesis

**Figure 9:**
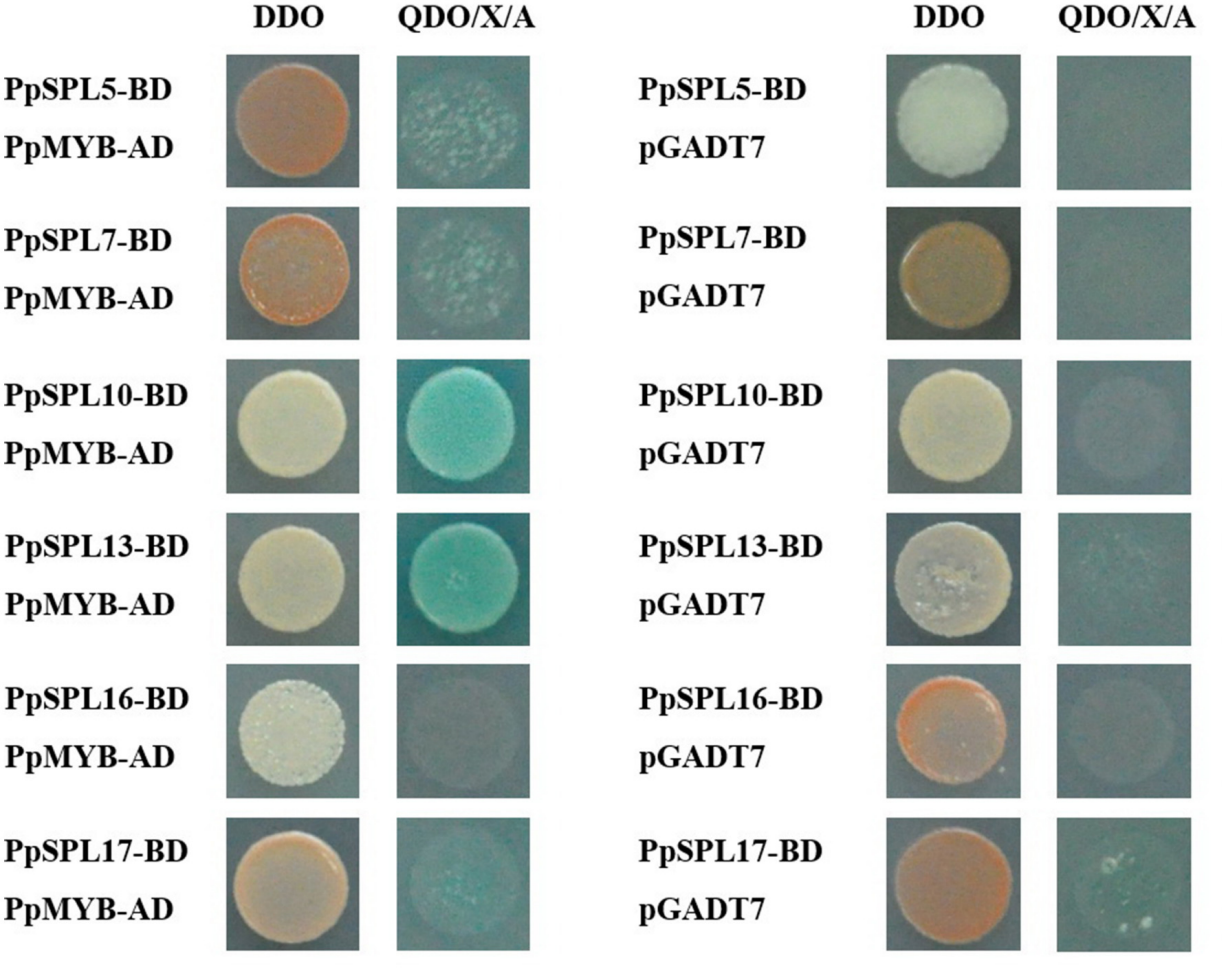
Yeast two-hybrid analysis of the physical interactions between PpSPL proteins and PpMYB10. The empty pGADT7 vector was used as a negative control.

In this study, *DFR* expression was slightly upregulated (<2-fold increase) during anthocyanin accumulation (Figure 5), and *NAC* genes were not considered. Thus, proteins encoded by genes that were downregulated after bags were removed from plants (e.g., *PpSPL2, PpSPL5, PpSPL7, PpSPL9, PpSPL10, PpSPL13, PpSPL16*, and *PpSPL17*) were fused to BD, while PpMYB10, PpbHLH3, and PpbHLH33 were fused to AD to test potential interactions in a yeast two-hybrid assay. Unfortunately, PpSPL2-BD, PpSPL9-BD, and PpSPL18-BD were autoactivated (data not shown). Our results indicated that PpSPL10 and PpSPL13 interacted with PpMYB10 (Figure 9), while there was no interaction between PpSPL and PpbHLH (Supplementary Figure 1).

## Discussion

### 11 *PpSPLs* were putative miR156 target genes in pear

Different family members of miR156 and *SPL* genes have been isolated in plants (Cardon et al., 1999; Yu et al., 2015). There are eight miR156 genes in the *A. thaliana* genome (i.e., miR156A, miR156B, miR156C, miR156D, miR156E, miR156F, miR156G, and miR156H). The expression patterns of these genes were highly tissue-specific. For example, miR156A was highly expressed in the first two pairs of leaves in seedlings, miR156B was specifically expressed in the central cylinder of the primary root primordial cells, and miR156D was highly expressed in seedlings (Yu et al., 2015). This variability in expression patterns suggests that the miR156 genes have diverse roles influencing plant growth and development.

Because of their crucial contribution to the regulation of a broad range of developmental processes, *SPL* genes have been subjected to genome-wide identification and analysis in diverse plant species. Consequently, 17, 27, 19, 19, and 15 *SPL* gene family members have been identified in *A. thaliana* (Cardon et al., 1999), apple (Li et al., 2013), grapevine (Wang et al., 2010), rice (Xie et al., 2006), and tomato (Salinas et al., 2012), respectively. Of the known *SPL* genes, only *SPL9* and *SPL10* in *A. thaliana* and *SPL1* in peach have been shown to participate in regulating anthocyanin biosynthesis (Gou et al., 2011; Zhou et al., 2015). There is currently no information regarding if specific miR156 and *SPL* gene family members are involved in regulating anthocyanin accumulation in pear.

In this study, we identified 26 miR156 members (Supplementary Tables S7 in Supplementary File 1), and 19 pear *PpSPL* genes (Supplementary Table S11 in Supplementary File 1). According to the phylogenetic tree, the SBP domains were clustered into seven distinct groups (i.e., G1–G7), which was similar to the results of a previous study on apple (Li et al., 2013). The *PpSPL* family members were more closely related to the *SPL* genes from eudicots, including apple, grape, tomato, and *A. thaliana*, than the rice *SPL* genes (i.e., monocotyledonous species). These findings imply that *SPL* genes in eudicots diverged more recently from a common ancestor than the *SPL* genes of monocots. In addition, 11 *PpSPL* genes are potentially regulated by miR156 (Supplementary Tables S10 in Supplementary File 1; Figure 3), which was similar to apple, where more than half of the *MdSBP* genes (15 out of 27) are targeted by miR156 (Li et al., 2013).

### Increased expression levels of *PpMYB10, PpCHS*, and *PpUFGT* were related to light-induced anthocyanin accumulation in pear

During the accumulation of anthocyanin after bags were removed from plants, the expression of most anthocyanin biosynthetic genes were upregulated. *PpCHS* and *PpUFGT* expression was especially upregulated at 3 DABR, with about 200- and 600-fold increases over expression levels at 0 DABR (Figure 5). Chalcone synthase catalyzes the first committed step of flavonoid and anthocyanin biosynthesis, and the *CHS* gene is considered to be a UV-B-responsive gene. Additionally, it is targeted by HY5, which is a bZIP transcription factor that mediates UV-B-induced gene expression changes downstream of UVR8 (Binkert et al., 2014). In our previous studies, *CHS* expression was induced by light (Qian et al., 2013) and methyl jasmonate (Qian et al., 2014b). Similar results were reported for other species, including apple (Ubi et al., 2006), Chinese bay berry (Niu et al., 2010), and *Petunia hybrida* (Koes et al., 1989). The UFGT enzyme catalyzes the production of stable anthocyanin by adding sugar moieties to the anthocyanin aglycone. The transcription of *UFGT* is one of the most rate-limiting steps in the anthocyanin biosynthesis pathway (Montefiori et al., 2011). In white grapes, the loss of fruit skin coloration is due to the absence of *UFGT* transcription (Boss et al., 1996). In litchi (*Litchi chinensis*), of the six genes encoding proteins associated with anthocyanin biosynthesis, the *UFGT* genes were the only ones whose transcript levels were significantly correlated with the pericarp anthocyanin content (Wei et al., 2011). These observations suggest that UFGT is required for anthocyanin accumulation.

Among transcription factors, the *PpMYB10* and *PpWD40* expression levels increased significantly, with peaks at 1 DABR (Figure 5). Additionally, the R2R3 MYB class of transcription factors is the dominant regulator of anthocyanin biosynthesis in several plant species, including pear (Feng et al., 2010), apple (Espley et al., 2009), grape (Kobayashi et al., 2004), and Chinese bayberry (Niu et al., 2010). Specific R2R3 MYBs were reported to activate the promoter regions of anthocyanin biosynthetic genes, such as *DFR* (Niu et al., 2010) and *UFGT* (Wang et al., 2014), thereby promoting anthocyanin accumulation. In Chinese sand pear, *PpMYB10* expression was considerably upregulated during anthocyanin biosynthesis in different developmental stages (Feng et al., 2010), following methyl jasmonate treatments (Qian et al., 2014b), and after postharvest irradiation (Qian et al., 2013). These findings, together with the results of the present study, indicate that upregulated *PpMYB10* expression is related to light-induced anthocyanin accumulation in pear. Interestingly, almost no anthocyanin concentration is detected in European pear under bagging treatment, and the expression of *PpMYB10* is not up-regulated as well (Qian et al., 2013), indicating that bagging treatment doesn't activate the light transduction pathway in European pear, or probably the regulation of *PpMYB10* expression from the down-stream transcription factors of light transduction pathway (such as HY5) is blocked due to certain factors. Thus, the transcription of *PpMYB10* is not triggered and the anthocyanin biosynthesis pathway is not activated.

### miR156-SPL module also responded during the light-induced anthocyanin biosynthesis in pear

During the biosynthesis of anthocyanin after bags were removed from plants, miR156 a/ba/g/s/sa abundance increased and peaked at 1–3 DABR (Figure 6). In contrast, we observed a decrease in the transcription levels of *PpSPL2, PpSPL5, PpSPL7, PpSPL9, PpSPL10, PpSPL13, PpSPL16, PpSPL17*, and *PpSPL18* (Figure 7). Additionally, *PpSPL9* and *PpSPL13* were determined to be homologs of *A. thaliana AtSPL9*, which regulates anthocyanin biosynthesis (Gou et al., 2011). In *A. thaliana*, increased miR156 activity promotes anthocyanin accumulation, whereas SPL activity is negatively correlated with anthocyanin content in wide-type plants and transgenic lines expressing Pro35S:MIR156 and Pro35S:MIM156 (Gou et al., 2011). Furthermore, during anthocyanin accumulation in *A. thaliana* plants treated with salt and mannitol, miR156 expression levels increased, while *SPL* expression was downregulated (Cui et al., 2014). In the red-fleshed peach cultivar “Dahongpao”, *SPL1* was highly expressed in white-fleshed young fruits, but expressed at low levels in red-fleshed ripening fruits (Zhou et al., 2015). These results indicate that miR156 expression is closely related to anthocyanin accumulation, while the expression of the target *SPL* genes is negatively correlated with anthocyanin biosynthesis. Concerned about the patterns anthocyanin accumulation and gene expression, we hypothesized the procedure of light-induced anthocyanin biosynthesis from up-stream to down-stream: Exposure to light increases the abundance of miR156 (peaked at 1 DABR), which degrades *SPL* genes (down-regulated at 1 DABR), resulting in the up-regulation of PpMYB10 (peaked at 1 DABR) and formation of the MYB/bHLH/WD40 complex, subsequently activates the expression of most structural genes including *PpCHS, PpCHI, PpF3H, PpANS*, and *PpUFGT* (peaked at 3 DABR). More proteins encoded by these genes are translated, catalyze the reactions of anthocyanin biosynthesis pathway, and finally lead to the accumulation of anthocyanin from 3 DABR to 6 DABR.

### PpMYB, PpbHLH, and PpWD40 could form a protein complex

The regulation of anthocyanin biosynthesis involves numerous transcription factors, including MYB, bHLH, and WD40, which form the MYB/bHLH/WD40 complex that mediates the expression of anthocyanin biosynthetic genes in diverse species, including *A. thaliana* (Broun, 2005), Chinese bayberry (Liu et al., 2013), and strawberry (Schaart et al., 2013). Among these transcription factors, bHLH acts as a bridge interacting with MYB and WD40 to form the complex. An earlier study involving apple revealed that there is no direct interaction between MYB and WD40 proteins (An et al., 2012). To the best of our knowledge, the current study is the first to examine the MYB/bHLH/WD40 complex in pear. Our yeast two-hybrid results indicated that PpMYB10 and PpWD40 interacted with PpbHLH3 and PpbHLH33, but not with each other. PpbHLH3 and PpbHLH33 were observed to interact with each other (Figure 8). Our findings imply that the MYB10/bHLH/WD40 transcription factor complex is conserved among plant species.

### PpSPL10 and PpSPL13 could form a heterodimer with PpMYB10

Our results revealed that PpSPL10 and PpSPL13 (orthologs of *A. thaliana* AtSPL9) interacted with PpMYB10 (Figure 9). We also observed that *DFR* expression was slightly upregulated (<2-fold increase) during anthocyanin accumulation (Figure 5). Therefore, the miR156–SPL9–DFR pathway, which responds to abiotic stresses in *A. thaliana* (Cui et al., 2014), may not be responsible for the light-induced accumulation of anthocyanin in pear. According to the previous results in *A. thaliana* (Gou et al., 2011), we hypothesize the involvement of miR156-*SPL* module in light-induced pear peel coloration and anthocyanin accumulation probably through the interaction between SPL and MYB10, which is the crucial part of MYB/bHLH/WD40 complex.

In conclusion, after bag removal, during the accumulation of anthocyanin in pear and up-regulation of genes related to anthocyanin biosynthesis, the expression of miR156 and its target *PpSPL* genes, including *PpSPL2, PpSPL5, PpSPL7, PpSPL9, PpSPL10, PpSPL13, PpSPL16, PpSPL17*, and *PpSPL18* were increased and decreased, respectively. In addition, three transcription factors MYB10, bHLH, and WD40 could form a protein complex, and PpSPL10 and PpSPL13 could also interact with PpMYB10. Our results provide some clues about the miR156-*SPL* module probably participating in light-induced red peel coloration in pear.

## Author contributions

MQ, JN, YT, and DZ designed the study; MQ, JN, and QN conducted the experiments; MQ, JN, QN, SB, LB, JL, YS, YT, and DZ analyzed the data; MQ, YT, and DZ wrote the article.

### Conflict of interest statement

The authors declare that the research was conducted in the absence of any commercial or financial relationships that could be construed as a potential conflict of interest. The reviewer JL declared a shared affiliation, though no other collaboration, with the authors LB and DZ to the handling Editor, who ensured that the process nevertheless met the standards of a fair and objective review.

## Supplementary material

Supplementary Figure S1 Yeast two-hybrid analysis of the physical interactions between PpSPL proteins and PpbHLH3, PpbHLH33, and PpWD40. The empty pGADT7 vector was used as a negative control.

Supplementary Table S1 Sequences of primers used to clone *PpSPLs*.

Supplementary Table S2 Primers for Q-PCR analysis of anthocyanin biosynthetic and regulatory genes.

Supplementary Table S3 Primers for Q-PCR analysis of *PpSPLs*.

Supplementary Table S4 qRT-PCR primer sequences used to amplify miR156.

Supplementary Table S5 Primers for yeast two-hybrid.

Supplementary Table S6 Distribution of sequencing reads from the library in pear.

Supplementary Table S7 Detailed list of homologous sequences for known miRNAs.

Supplementary Table S8 Detailed list of novel and candidate miRNAs found in “Meirensu” pear.

Supplementary Table S9 Targets of pear miRNAs (or miRNA families; detailed list).

Supplementary Table S10 GO annotation of target genes.

Supplementary Table S11 *PpSPL* gene family in pear.

Supplementary Table S12 General information of *SBP-box* genes selected for phylogenetic analysis.

## Abbreviations

AbA: aureobasidin A
ANS: anthocyanidin synthase
CHI: chalcone isomerase
CHS: chalcone synthase
DABR: days after bag removal
DDO: double dropout
DFR: dihydroflavonol 4-reductase
F3H: flavanone 3-hydroxylase
GO: Gene Ontology
HMM: hidden Markov model
PAL: phenylalanine ammonia lyase
QDO: quadruple dropout
qPCR: quantitative real-time polymerase chain reaction
SD: synthetically defined
SPL: SQUAMOSA PROMOTER BINDING PROTEIN-LIKE
UFGT: UDP-glucose:flavonoid 3-O-glucosyltransferase
X-α-Gal: 5-bromo-4-chloro-3-indolyl α-D-galactopyranoside
5′-RACE: 5′-Rapid amplification of cDNA ends.
