# Supplementary figures and images for: Response of miR156-SPL Module during the Red Peel Coloration of Bagging-Treated Chinese Sand Pear (Pyrus pyrifolia Nakai)
Source: Front Physiol. 2017 Aug 7;8:550. doi: 10.3389/fphys.2017.00550 (PMC5545762; doi:10.3389/fphys.2017.00550)

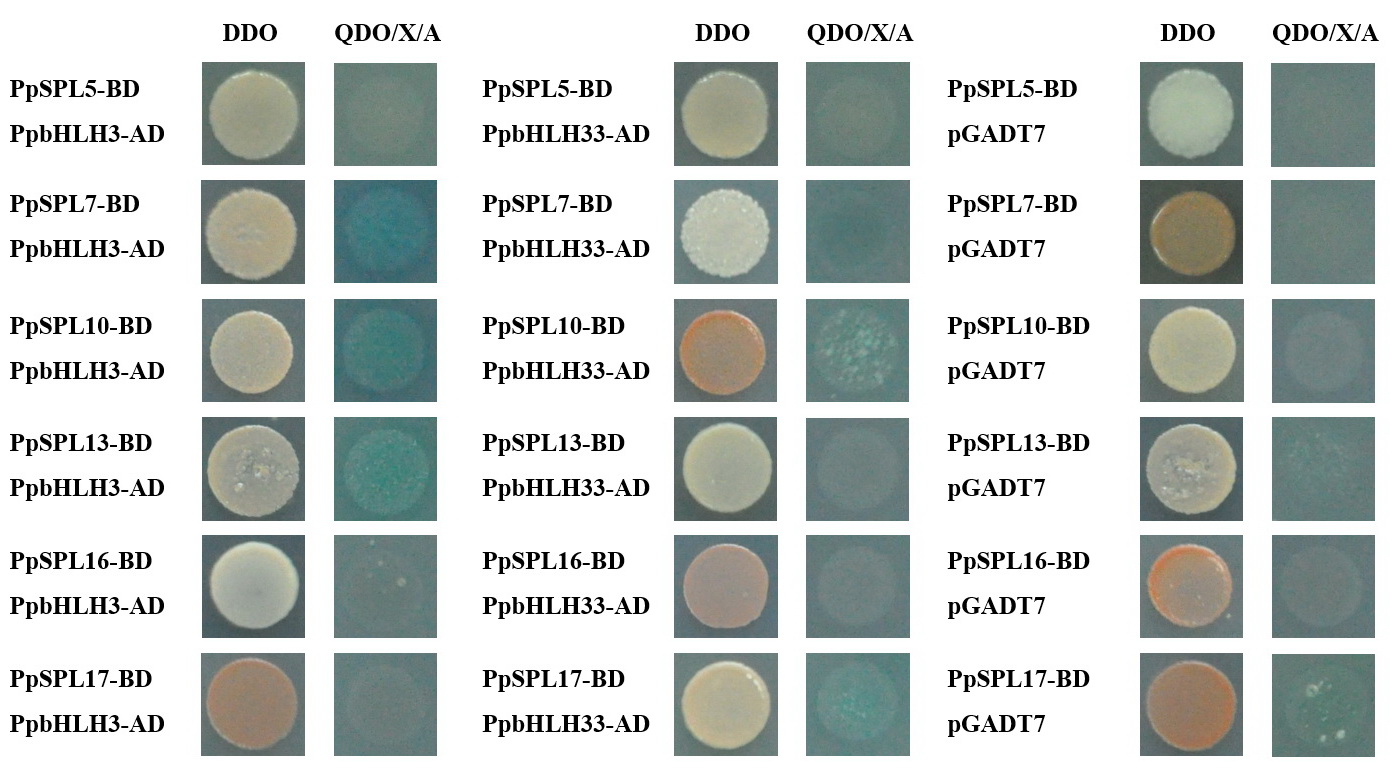

Supplement: Supplementary Figure S1 — Yeast two-hybrid analysis of the physical interactions between PpSPL proteins and PpbHLH3, PpbHLH33, and PpWD40. The empty pGADT7 vector was used as a negative control. [file Image1.JPEG]
